# Factors influencing length of stay in orthopedic Class I incision surgery: development and validation of a nomogram using 31,248 patient records

**DOI:** 10.3389/fmed.2025.1689556

**Published:** 2026-01-12

**Authors:** Binbin Fu, Chi Tong, Lingli Wang, Zhiwen Zhao, Yuehui Huang, Ruiping Lai, Hanlin Liao, Duoshuang Xie

**Affiliations:** 1 Department of Healthcare-Associated Infection Management, Taihe Hospital, Hubei University of Medicine, Shiyan, Hubei, China; 2 Department of Emergency Medicine, Taihe Hospital, Hubei University of Medicine, Shiyan, Hubei, China; 3 Department of Healthcare-Associated Infection Management, The Second People's Hospital of Changzhi, Changzhi, Shanxi, China; 4 Department of Medical Education, Taihe Hospital, Hubei University of Medicine, Shiyan, Hubei, China

**Keywords:** Class I incision, length of stay, nomogram, orthopedic surgery, surgical grade

## Abstract

**Objective:**

Prolonged length of stay (LOS) following orthopedic surgery places a significant strain on healthcare systems. However, effective tools for predicting LOS in patients undergoing clean (Class I) orthopedic surgery are lacking. This study aims to identify factors influencing length of stay in orthopedic Class I incision surgery and construct a predictive nomogram based on these factors.

**Methods:**

Retrospective analysis of patients undergoing orthopedic Class I incision surgery in Taihe Hospital from January 1, 2018 to October 31, 2023. Patients meeting the inclusion criteria were enrolled. Using prolonged length of stay (LOS > 7 days) as the primary outcome, we performed univariate analysis followed by binary logistic regression to identify risk factors. An individual nomogram was developed using R 4.3.3.

**Results:**

31,248 patients were ultimately included, with 20,419 (65.34%) patients demonstrating prolonged length of stay (LOS > 7 days). The results of binary logistic regression show that the independent risk factors for prolonged LOS (LOS > 7 days) in patients undergoing orthopedic Class I incision surgery were: age, surgical duration, surgical grade, American Society of Anesthesiologists’ Physical Status Classification System (ASA PS), antibiotic use, combined antibiotic, and blood potassium(K), sodium (Na), magnesium(Mg) and calcium(Ca) concentrations. Validation using the receiver operating characteristic (ROC) curve showed that the nomogram had an area under the curve (AUC) of 0.846 (95% CI: 0.841–0.850), demonstrating good accuracy. The bootstrap method was used to repeatedly sample 1,000 times to verify the nomogram. The mean absolute error of the calibration curve was 0.003, indicating that the calibration curve fits well with the ideal curve. Decision curve analysis showed a significantly greater net benefit of the nomogram.

**Conclusion:**

The developed nomogram accurately predicts prolonged hospitalization risk in orthopedic patients with Class I incisions, integrating key determinants including age, surgical complexity, physiological status, and electrolyte levels. This tool demonstrates robust performance and offers tangible clinical utility for optimizing resource allocation and guiding personalized perioperative management.

## 1 Introduction

China is currently experiencing significant transformations in its demographic structure. Projections indicate that by 2040, the population aged 60 and above will reach 402 million, constituting 28% of the total population (1). Elderly individuals and patients with chronic comorbidities represent the primary demographic requiring medical services (2). The strain on China’s healthcare resources is substantial, attributable to two major factors: Firstly, the high population density in urban areas, coupled with rapid urbanization, has placed considerable pressure on medical infrastructure (3); Secondly, the incidence of orthopedic diseases has been progressively increasing due to the combined effects of socioeconomic development, expanded transportation networks, and other contributing factors, thereby demanding greater allocation of medical resources (4). In this context, reducing the length of stays (LOS) emerges as a viable strategy to mitigate the scarcity of medical resources.

Extensive research has demonstrated that reducing LOS can lead to multiple benefits, including decreased hospitalization costs, reduced risk of nosocomial infections, and improved patient satisfaction (5, 6). However, there is currently a lack of effective predictive tools for estimating LOS specifically in orthopedic patients with clean (Class I) incisions. The LOS often reflects disease severity, and identifying predictive variables for LOS in orthopedic surgery could significantly contribute to the development of preventive and therapeutic strategies (7). According to the “Technical Guidelines for Prevention and Control of Surgical Site Infections,” surgical incisions are classified into four categories based on the degree of microbial contamination. Among these, Class I incisions refer to those that do not enter infected or inflamed areas and do not involve communication with hollow viscera such as the respiratory, alimentary, or genitourinary tracts. Notably, Class I incisions constitute the majority of orthopedic surgical procedures. Although their infection risk is relatively low, the occurrence of surgical site infections (SSI) can still significantly prolong LOS (6).

To eliminate the potential confounding effects of wound contamination on SSI incidence and LOS, this study specifically focuses on orthopedic surgery patients with Class I incisions. Through a comprehensive analysis of patient demographics, surgical parameters, antibiotic usage, and preoperative electrolyte level, this study aims to identify the key factors influencing LOS and to develop a personalized nomogram prediction model based on these factors. This tool is intended to aid in the early identification of high-risk patients, guide targeted perioperative management, and support the rational allocation of medical resources.

## 2 Methods

### 2.1 Study design and participants

This study was a single-center, retrospective observational study aimed at identifying factors influencing the length of stay (LOS) in patients undergoing orthopedic Class I incision surgery, and to develop and validate a prediction model for identifying high-risk patients for prolonged LOS (LOS > 7 days) after surgery. This study was conducted at Taihe Hospital, a major tertiary comprehensive medical center affiliated with Hubei University of Medicine in Shiyan City, Hubei Province, China.

Patient data were retrospectively collected from the hospital information system (HIS) for orthopedic cases undergoing Class I incision surgeries between January 1, 2018 and October 31, 2023. To minimize potential confounding effects of multiple surgical procedures on LOS, the study exclusively included patients who underwent a single surgical procedure during their hospitalization. For patients with two or more separate hospitalizations during the study period, each admission was treated as an independent case. The exclusion criteria were as follows: (1) patients undergoing multiple surgical procedures within a single hospitalization; (2) cases with incomplete or missing essential clinical data. Initial screening included 31,363 patients. After excluding 115 cases with missing key variables (e.g., surgery grade), a total of 31,248 patients were ultimately included in the analysis. Each hospitalization had a unique admission ID, and multiple hospitalizations of the same patient had different IDs. All data were accurately matched and integrated across systems via this unique admission number.

The research data were obtained from the HIS, which consists of a basic information database and a laboratory testing database. The collected variables included: (1) Demographic characteristics: sex, age, hospitalization number and LOS; (2) Clinical parameters: operation time, antibiotic use, ASA PS (American Society of Anesthesiologists’ Physical Status Classification System), surgery grade; (3) Preoperative electrolyte levels: potassium (K), sodium (Na), magnesium (Mg), phosphorus (P), and calcium (Ca) concentrations (8, 9). It should be particularly noted that while hospitalized patients may undergo multiple preoperative and postoperative examinations, this study exclusively selected the last preoperative laboratory test results for analysis. Antibiotic use was not categorized as prophylactic or therapeutic.

The statistical analysis followed these steps: First, univariate analysis was performed to screen for variables significantly associated (*p* < 0.05) with prolonged LOS. Subsequently, variables with statistical significance were included in a multivariate logistic regression analysis to identify independent risk factors. Based on the final independent factors, a nomogram prediction model was constructed. The model performance was validated, and decision curve analysis was used to evaluate the clinical utility and net benefit of the model.

### 2.2 Variable definitions and measurements

The following details the definitions and classification criteria for two key graded variables in this study: surgical grade and ASA PS. According to the Chinese “Administrative Measures for Hierarchical Surgery Management in Medical Institutions,” surgical procedures are classified into four hierarchical levels, from Level I to Level IV, in ascending order based on their risk, technical complexity, resource consumption, and ethical considerations. Level I procedures refer to those with relatively low risk, simple processes, and low technical difficulty. In contrast, Level IV procedures are characterized by high risk, complex processes, significant technical difficulty, and potential for high resource consumption or major ethical implications (10). ASA PS characterizes a patient’s preoperative physical status. It comprises categories 1 through 5, which represent increasing levels of patient impairment-from a “normal healthy patient” (ASA PS 1) to a “moribund patient who is not expected to survive without the operation” (ASA PS 5). An additional Category 6 is designated for a declared brain-dead organ donor (11). In this study, all enrolled patients had ASA PS ratings ranging from 1 to 4.

### 2.3 Statistical methods

For categorical variables, we used frequencies and percentages to describe them. Chi-squared test was used to examine the differences in LOS among categorical variables. Quantitative variables were skewed after normality testing, we described them using median and interquartile range (IQR). Mann–Whitney U test was carried out to test differences. Independent variables with *p* < 0.05 were included in the binary logistic regression analysis (Forward: LR). We used IBM SPSS Statistics (Version 25.0) for descriptive analysis, univariate analysis, and binary logistic regression analysis. Construct a nomogram based on the results of logistic regression. The model’s performance was evaluated by assessing its discrimination using the receiver operating characteristic (ROC) curve and the area under the curve (AUC), and its calibration with a calibration curve. Decision curve analysis (DCA) reflected the net benefit of the model for patients. Constructed nomogram and model validation using R programming language and environment. *p* < 0.05 (two-sides) was considered as statistically significant.

## 3 Results

### 3.1 Characteristics of participants and group comparison of length of stay

As shown in Table 1, a total of 31,248 cases of orthopedic Class I incision surgery were included in this study. 17,319 (55.424%) were males, 13,929 (44.576%) were females. The median age was 50.000 (35.000 ~ 60.000) years. 25,789 (82.530%) cases had an ASA PS of 2. 13,268 (42.460%) cases with surgical grade 2. 20,189 (64.609%) cases were treated with antibiotics. The median and interquartile intervals of the test data were shown in Table 1. Gender, surgical grade, ASA PS, using antibiotics, combination use of antibiotics, age and surgical duration were associated with the LOS. The Mann–Whitney U test results showed that all test items were related to LOS.

**Table 1:** Patient characteristics and comparison of length of stay (LOS) among different groups (*n* = 31,248).

Variables	*N* (%) 31,248	Length of hospital stay [*N* (%)/IQR]		*p*
		≤7 10,829 (34.655)	>7 20,419 (65.345)	
Gender				0.008
Male	17,319 (55.424)	6,113 (19.563)	11,206 (35.861)	
Female	13,929 (44.576)	4,716 (15.092)	9,213 (29.483)	
Surgical grade^a^				<0.001
1	1,318 (4.218)	1,051 (3.360)	7,588 (24.283)	
2	13,268 (42.460)	5,680 (18.177)	6,657 (21.304)	
3	9,333 (29.868)	2,267 (7.255)	7,066 (22.613)	
4	7,329 (23.454)	1831 (5.860)	5,498 (17.595)	
ASA PS^b^				<0.001
1	3,516 (11.252)	1968 (6.298)	1,548 (4.954)	
2	25,789 (82.530)	8,502 (27.208)	17,287 (55.322)	
3	1844 (5.901)	326 (1.043)	1,518 (4.858)	
4	99 (0.317)	33 (0.106)	66 (0.211)	
Antibiotic use				<0.001
No	11,059 (35.391)	6,917 (22.136)	4,142 (13.255)	
Yes	20,189 (64.609)	3,912 (12.519)	16,277 (52.090)	
Combination use of antibiotics				<0.001
No	24,683 (78.991)	9,410 (30.114)	15,273 (48.877)	
Yes	6,565 (21.009)	1,419 (4.541)	5,146 (16.468)	
Age (years)	31,248 (100.000)	44.000 (24.000 ~ 56.000)	52.000 (41.000 ~ 62.000)	<0.001
Surgical duration (hours)	31,248 (100.000)	1.000 (0.420 ~ 1.500)	2.000 (1.330 ~ 2.750)	<0.001
K	31,248 (100.000)	4.110 (3.900 ~ 4.330)	4.010 (3.800 ~ 4.230)	<0.001
Na	31,248 (100.000)	141.100 (139.615 ~ 142.690)	141.400 (139.800 ~ 144.410)	<0.001
Mg	31,248 (100.000)	0.860 (0.810 ~ 0.910)	0.850 (0.800 ~ 0.900)	<0.001
P	31,248 (100.000)	1.120 (0.970 ~ 1.325)	1.070 (0.930 ~ 1.220)	<0.001
Ca	31,248 (100.000)	2.330 (2.250 ~ 2.420)	2.270 (2.190 ~ 2.360)	<0.001

^a^Surgical grade was defined using the Chinese “Administrative Measures for Hierarchical Surgery Management in Medical Institutions” (Level I-IV, with I indicating the lowest complexity). ^b^American Society of Anesthesiologists Physical Status classification, with higher scores representing greater impairment.

### 3.2 Binary logistic regression analysis for prolonged LOS (LOS >7 days)

The researchers included all significant variables in univariate binary logistic regression and multivariate binary logistic regression, respectively. As shown in Table 2, after adjusting for gender, surgical grade, ASA PS, whether antibiotics were used, and whether antibiotics were used in combination, researchers found that the risk of LOS >1 week in patients undergoing level 2 surgery was 2.837 times that of patients undergoing level 1 surgery, the risk in patients undergoing level 3 surgery was 4.028 times that of patients undergoing level 1 surgery, the risk in patients undergoing level 4 surgery was 2.061 times that of patients undergoing level 1 surgery. The risk of LOS >1 week in patients with an ASA PS of 2 was 1.282 times that of patients with an ASA PS of 1, the risk in patients with an ASA PS of 3 was 1.526 times that of patients with an ASA PS of 1, the risk in patients with an ASA PS of 4 was 1.621 times that of patients with an ASA PS of 1. Patients who had used antibiotics were 3.156 times more likely to report a LOS of more than 1 week than those who had not used antibiotics. Patients who had used antibiotics in combination were 0.866 times more likely to report a LOS of more than 1 week than those who had not used antibiotics in combination. Among continuous variables, factors that were directly proportional to LOS included age, surgical duration and Na. K, Mg and Ca were inversely proportional to LOS.

**Table 2:** Univariate and multivariate logistic regression analysis for prolonged length of stay (LOS > 7 days).

Variables	Univariate OR (95%CI)	*p*	Multivariate OR (95%CI)	*p*
Gender (female as reference)				
Male	0.920 (0.874 ~ 0.969)	0.002	1.026 (0.965 ~ 1.090)	0.416
Surgical grade (1 as reference)^a^				
2	5.332 (4.562 ~ 6.233)	<0.001	2.837 (2.427 ~ 3.316)	<0.001
3	12.236 (10.427 ~ 14.358)	<0.001	4.028 (3.427 ~ 4.735)	<0.001
4	11.764 (10.000 ~ 13.841)	<0.001	2.061 (1.741 ~ 2.441)	<0.001
ASA PS (1 as reference)^b^				
2	2.603 (2.404 ~ 2.817)	<0.001	1.282 (1.167 ~ 1.407)	<0.001
3	6.060 (5.216 ~ 7.040)	<0.001	1.526 (1.277 ~ 1.824)	<0.001
4	2.582 (1.651 ~ 4.037)	<0.001	1.621 (0.934 ~ 2.813)	0.086
Antibiotic use (not using antibiotics as reference)				
Using antibiotics	7.071 (6.679 ~ 7.485)	<0.001	3.156 (2.945 ~ 3.383)	<0.001
Combination use of antibiotics (not combined with antibiotics as reference)				
Combined with antibiotics	2.192 (2.046 ~ 2.347)	<0.001	0.866 (0.794 ~ 0.942)	0.001
Age (years)	1.027 (1.025 ~ 1.028)	<0.001	1.020 (1.018 ~ 1.022)	<0.001
Surgical duration (hours)	3.351 (3.230 ~ 3.475)	<0.001	2.678 (2.584 ~ 2.775)	<0.001
K	0.455 (0.423 ~ 0.490)	<0.001	0.686 (0.629 ~ 0.748)	<0.001
Na	1.023 (1.013 ~ 1.034)	<0.001	1.017 (1.004 ~ 1.029)	0.007
Mg	0.158 (0.117 ~ 0.212)	<0.001	0.476 (0.338 ~ 0.671)	<0.001
P	0.310 (0.280 ~ 0.343)	<0.001	1.137 (0.995 ~ 1.298)	0.059
Ca	0.030 (0.024 ~ 0.037)	<0.001	0.220 (0.171 ~ 0.282)	<0.001

^a^Surgical grade was defined using the Chinese “Administrative Measures for Hierarchical Surgery Management in Medical Institutions” (Level I–IV, with I indicating the lowest complexity). ^b^American Society of Anesthesiologists Physical Status classification, with higher scores representing greater impairment.

### 3.3 Building nomogram model

**Figure 1:**
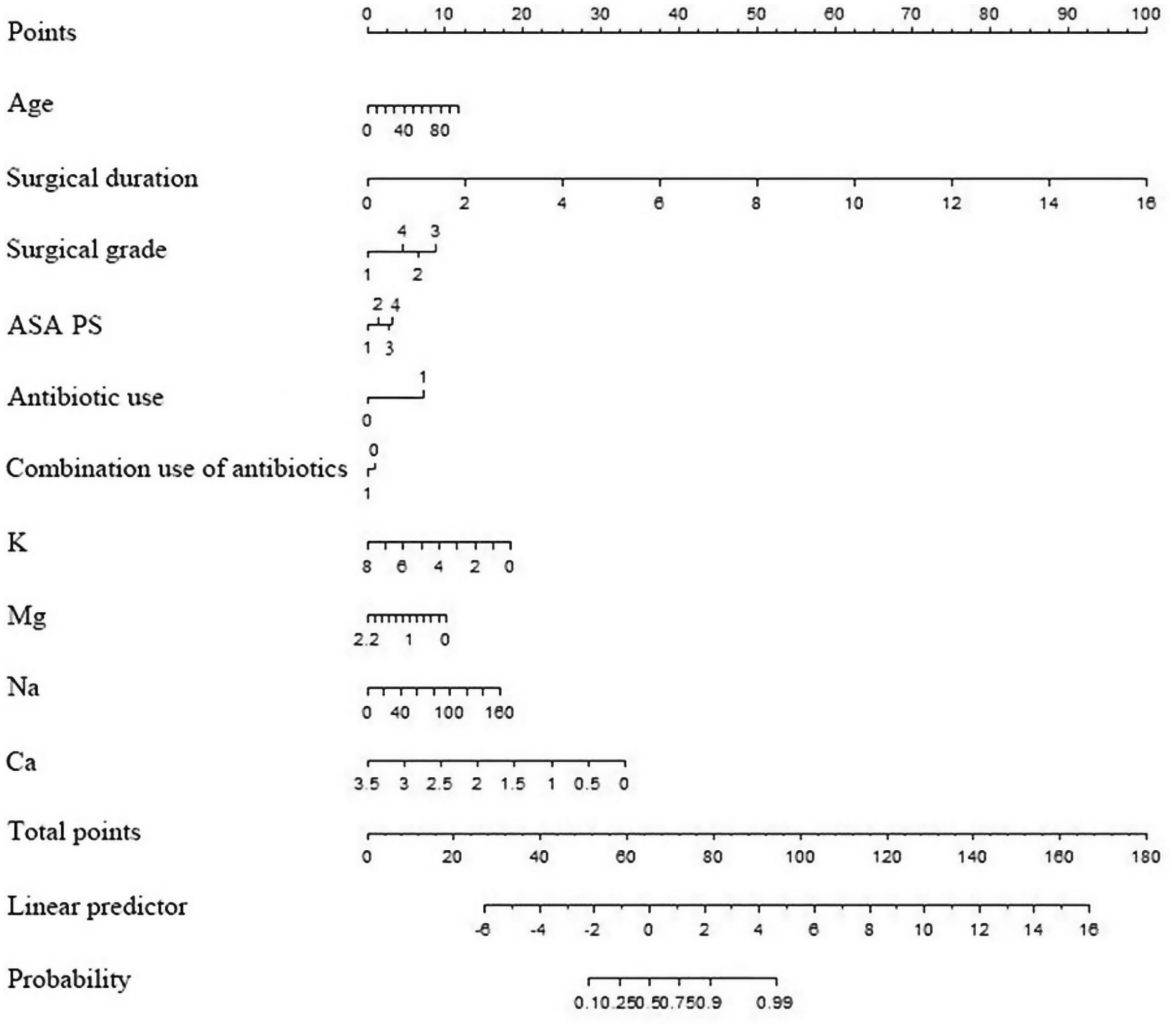
Nomogram for predicting the length of stay in patients undergoing orthopedic Class I incision surgery.

**Figure 2:**
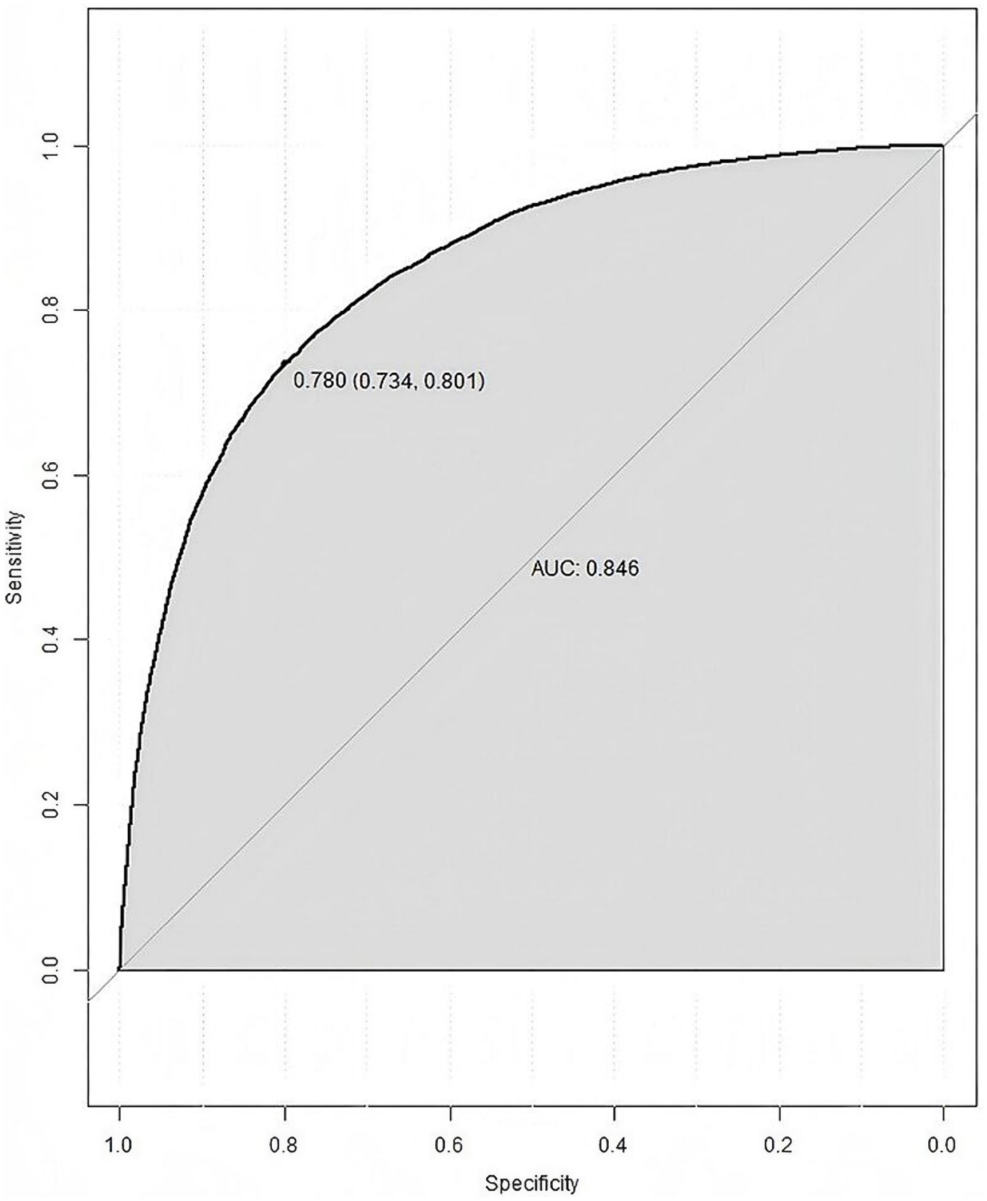
ROC curve of nomogram prediction model.

**Figure 3:**
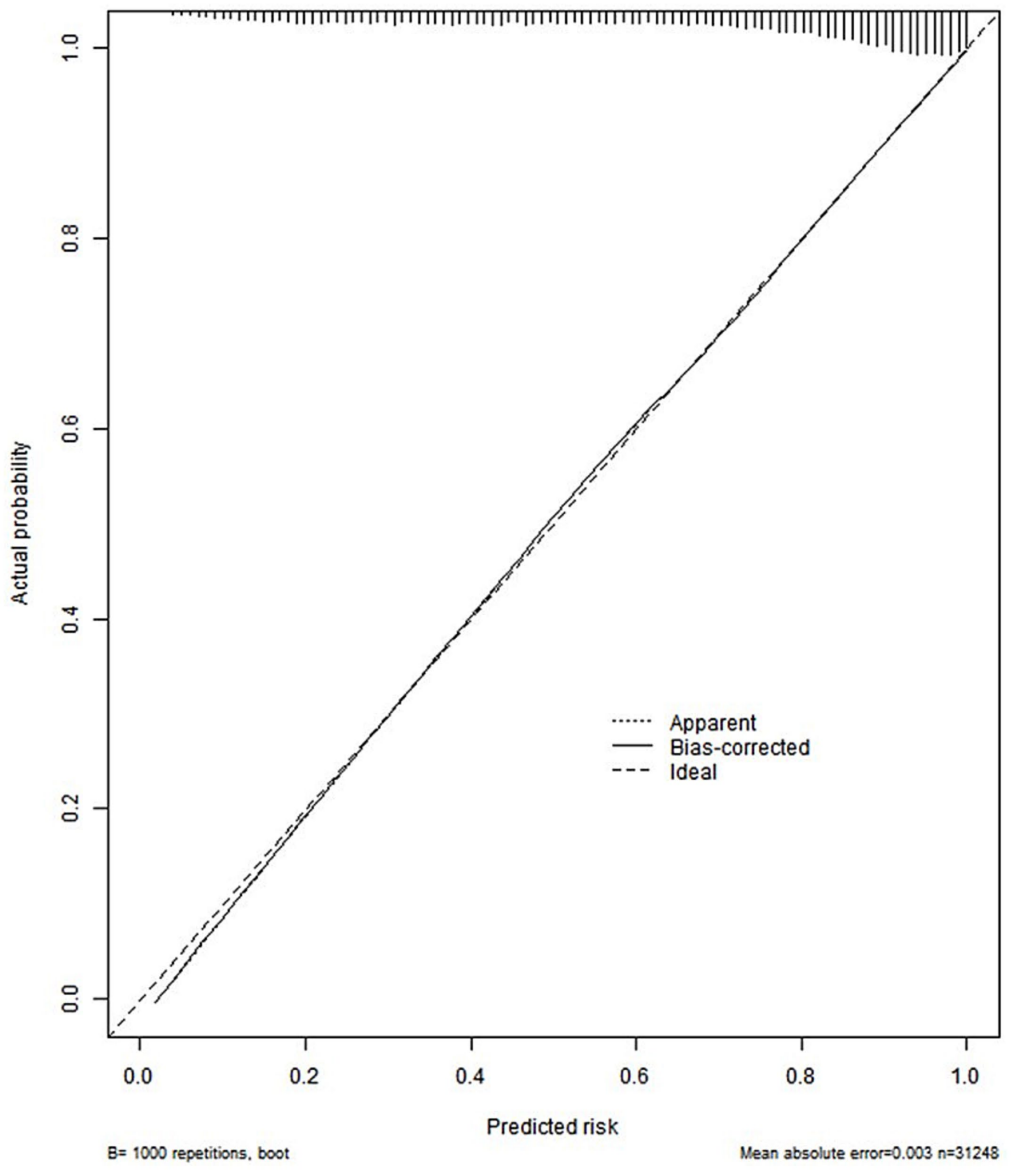
Bootstrap method validation nomogram curve.

**Figure 4:**
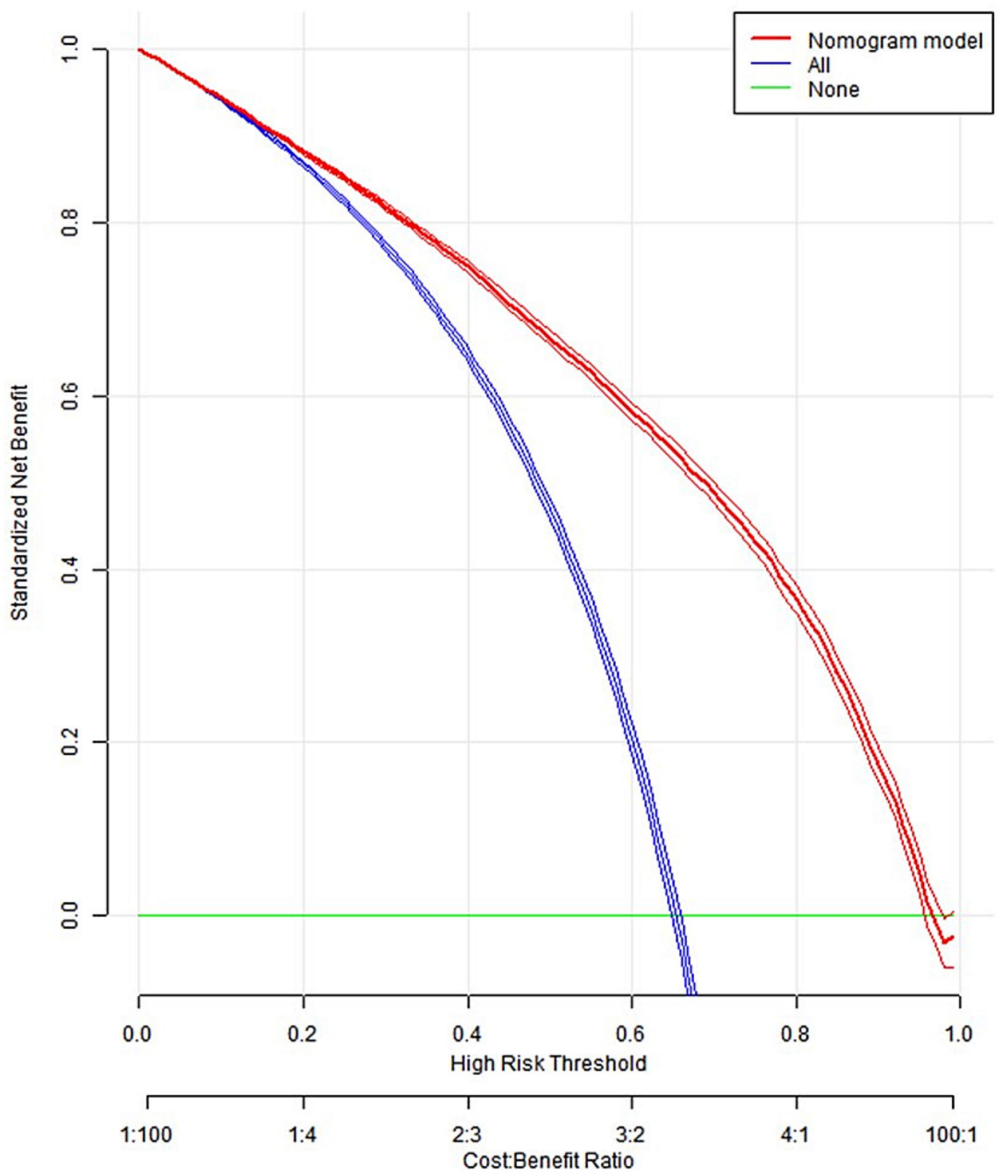
Clinical decision curve of nomogram model.

Constructed a nomogram model using R. The value on the horizontal axis of each indicator in the nomogram was plotted perpendicular to the focus of the value on the scoring scale to obtain a score. The sum of the values of all indicators was the risk value of poor prognosis for the patient. The specific results were shown in Figure 1. The ROC curve validation nomogram model showed AUC = 0.846, 95% CI: 0.841 ~ 0.850, reflecting the good accuracy of the nomogram model. The specific results were shown in Figure 2. The bootstrap method was used to repeatedly sample 1,000 times to verify the nomogram. The mean absolute error of the calibration curve was 0.003, indicating that the calibration curve fits well with the ideal curve. The specific results were shown in Figure 3. Decision curve analysis showed a significantly greater net benefit of the nomogram (Figure 4). In addition, 9,374 patients (30% of all patients) were used for external validation to test the nomogram. The AUC was 0.843 (95% CI: 0.835–0.851), reflecting good accuracy of the nomogram. The complete external validation results are provided in the Appendix.

## 4 Discussion

This study investigates the influencing factors of LOS for Chinese orthopedic patients undergoing Class I incision surgery. Currently, there is relatively limited research on LOS both domestically and internationally. The average LOS for orthopedic patients undergoing Class I incision surgery in this study was 11.5 ± 7.7 days, which is consistent with the average LOS for hip fractures and pelvic fractures reported in domestic and international studies (12, 13). The factors affecting the LOS of patients undergoing Class I incision surgery can exclude the influence of contaminated wounds, making these factors relatively easier to identify. To analyze the influencing factors of LOS for orthopedic Class I incision surgery patients, we constructed a personalized nomogram model based on the aforementioned factors and conducted this study.

First, we performed univariate analysis on the factors that might lead to prolonged LOS for orthopedic Class I incision surgery patients. Statistically significant differences were observed between the groups with LOS of ≤7 days and >7 days in terms of gender, surgical grade, ASA PS, antibiotic use, combined antibiotic use, age, surgical duration, K, Na, Mg, P, and *Ca.* To further clarify which of these influencing factors are truly independent risk factors, we conducted a logistic regression analysis with prolonged LOS (LOS > 7 days) as the dependent variable and the aforementioned statistically significant indicators as independent variables. The final factors identified as leading to prolonged LOS (LOS > 7 days) were: age, surgical duration, surgical grade, ASA PS, antibiotic use, combined antibiotic use, K, Na, Mg and *Ca.*

Age is directly proportional to the LOS. Elderly patients face an increased risk of pneumonia and falls due to decreased muscle mass and function, as well as prolonged bed rest. Additionally, the risk of frailty increases with age (14). Approximately 30 to 50% of elderly patients are in a weakened state, and the LOS for these patients increases by 15 to 60% (15). Furthermore, the nutritional status of elderly individuals is generally poor, and malnutrition is also a significant risk factor for prolonged hospitalization (16).

The duration of surgery is directly proportional to the LOS. On the one hand, longer surgical durations often indicate more complex patient conditions, necessitating extended LOS. On the other hand, prolonged surgical times increase the risk of postoperative surgical site infections, which are independent risk factors for extended LOS (17).

Except for level four surgeries, higher surgical levels are associated with longer LOS. According to the Chinese Medical Institution Surgical Grading Management Measures, higher-level surgeries entail greater risks and difficulties. Surgical risks include anesthesia risks, major surgical complications risks, etc. Surgical difficulty encompasses factors such as the complexity of the procedure, patient condition, and surgical duration. Consequently, higher surgical levels correlate with longer LOS. Level four orthopedic surgeries, primarily spinal surgeries, involve nerve-related risks (18). However, these surgeries are generally minimally invasive, resulting in shorter LOS.

The ASA PS is directly proportional to the LOS. Studies have shown that as the ASA PS increases, so do the risks of intraoperative blood loss, transfusion volume, postoperative complications, and infections (19).

The use of antibiotics is a risk factor for extended LOS. Antibiotics are categorized into prophylactic and therapeutic uses. According to the Guiding Principles of Clinical Application of Antibacterials (2015 Edition) (20), prophylactic antibiotics are not typically used for Class I incisions unless the surgery involves a large scope, prolonged duration, implantation of foreign bodies, or high-risk factors such as advanced age, diabetes, immune dysfunction, or malnutrition. These factors are all associated with prolonged LOS. However, the combination use of antibiotics can shorten LOS, likely due to a broader antimicrobial spectrum, enhanced efficacy, and reduced development of drug-resistant bacteria (21).

Serum concentrations of cations capable of binding acid radicals (e.g., K^+^, Mg^2+^, Ca^2+^) exhibit a significant negative correlation with LOS in fracture patients. This association may involve two mechanisms: First, these cations reflect systemic alkalization status (positively correlated with blood alkalinity), while an acidic microenvironment has been shown to suppress osteoblast activity and enhance osteoclast activation, thereby disrupting bone metabolic homeostasis (22, 23). Second, these ions exert direct osteoprotective effects. For instance, potassium reduces calcium excretion (24), and potassium bicarbonate lowers urinary calcium levels, thereby positively influencing bone turnover and calcium excretion (25). Epidemiological studies have confirmed that high potassium intake observed in diets rich in fruits and vegetables is positively correlated with femoral neck bone mineral density in adult women, while showing a negative correlation with biochemical markers of bone turnover (26). Therefore, for hospitalized fracture patients, it is recommended to maintain serum potassium levels at the upper limit of the normal reference range through dietary interventions, with priority given to potassium-rich foods to optimize the bone repair microenvironment.

Calcium, as an essential nutrient, plays a pivotal role in skeletal development, growth, and structural maintenance, while also participating in the regulation of cellular cytoskeletal stability mechanisms (27). Evidence-based medical studies demonstrate a significant positive correlation between insufficient dietary calcium intake and elevated fracture risk (28). Increased dietary calcium consumption or calcium supplementation has been shown to effectively enhance total body bone mineral density (29, 30), and reduce overall fracture incidence (31). Notably, under conditions of complete immobilization (as observed in post-traumatic cast fixation patients), calcium resorption from bone tissue occurs, leading to accelerated bone mass depletion (32). Based on these findings, clinical guidelines recommend implementing calcium supplementation protocols combined with high-calcium dietary regimens for fracture patients during hospitalization to maintain bone metabolic homeostasis.

Magnesium is one of the most essential elements in the human body. Approximately 50–60% of the body’s magnesium is distributed in bone tissue, playing a crucial role in mineral metabolism, bone homeostasis maintenance, osteocyte function regulation, and the growth and formation of hydroxyapatite crystals (33). Magnesium deficiency affects bone health through both direct and indirect pathways: the direct effects include reduced bone hardness, increased osteoclast activity, and inhibited osteoblast function; while the indirect effects involve interference with parathyroid hormone (PTH) and vitamin D metabolism, promoting inflammatory responses and oxidative stress, ultimately leading to bone loss (34). PTH and 1,25(OH)2D3 are the primary regulators of calcium homeostasis. Hypomagnesemia not only inhibits PTH secretion but may also reduce target organ sensitivity to circulating PTH, resulting in biochemical characteristics similar to primary hypoparathyroidism. Studies have shown that magnesium supplementation can effectively correct PTH and 1,25(OH)2D3 levels in postmenopausal osteoporotic women. Animal experiments have further confirmed that magnesium deficiency leads to increased bone fragility and significantly reduced mechanical properties (35). Our findings are consistent with these observations, demonstrating a positive correlation between higher serum magnesium concentrations and shorter LOS. However, it is noteworthy that excessively high magnesium concentrations may adversely affect bone metabolism and parathyroid function. *In vitro* experiments have confirmed that a high magnesium environment inhibits osteoblast differentiation and mineralization processes (36). Therefore, it is recommended to maintain serum magnesium concentrations at the upper limit of the normal reference range for fracture patients during hospitalization.

Serum sodium is a key electrolyte responsible for maintaining extracellular osmotic pressure and water-electrolyte balance, and its homeostasis is fundamental for ensuring normal cellular function and internal environment stability (37). This study identified elevated preoperative serum sodium concentration as an independent risk factor for prolonged length of hospital stay, suggesting that disruption of this homeostasis is closely associated with unfavorable postoperative recovery. This relationship may be attributed to the fact that hypernatremia often serves as a marker of underlying dehydration or endocrine dysfunction, conditions that can impair tissue perfusion and increase the risk of complications (38). Simultaneously, it also reflects a greater comorbidity burden and diminished physiological reserve in patients, commonly associated with chronic conditions such as heart failure, chronic kidney disease, and uncontrolled diabetes, which are frequently accompanied by disturbances in water and electrolyte metabolism (39–41). Therefore, preoperative serum sodium should be considered an effective risk stratification indicator, and active management of sodium homeostasis during the perioperative period in these patients is of significant clinical importance for controlling complications and reducing the length of hospital stay.

When contextualizing our findings within the existing literature, consistencies and divergences emerge that help delineate the specific contributions of this study. Previous studies have demonstrated that the LOS for orthopedic patients is influenced by multiple factors. Research by Rhee et al., which included 10,123 total hip arthroplasty (THA) and 17,243 total knee arthroplasty (TKA) patients, used linear regression models and identified independent risk factors for prolonged postoperative LOS, including comorbidities such as myocardial infarction (THA: 0.25, 95% CI: 0.17–0.34; TKA: 0.35, 95% CI: 0.28–0.41), intraoperative blood transfusion (THA: 0.28, 95% CI: 0.25–0.30; TKA: 0.32, 95% CI: 0.30–0.34), advanced age (60–69 years: THA: 0.07, 95% CI: 0.05–0.09; TKA: 0.03, 95% CI: 0.01–0.04; 70–79 years: THA: 0.18, 95% CI: 0.16–0.21; TKA: 0.10, 95% CI: 0.09–0.12; ≥80 years: THA: 0.37, 95% CI: 0.34–0.40; TKA: 0.23, 95% CI: 0.21–0.25), and female sex (THA: 0.09, 95% CI: 0.07–0.11; TKA: 0.08, 95% CI: 0.07–0.09). The adjusted *R*^2^ values were 0.375 for THA and 0.361 for TKA, indicating good model explanatory power (9).

Another large-scale study by Chona et al., utilizing the ACS-NSQIP database and encompassing 49,778 trauma orthopedic patients, employed negative binomial regression to develop a LOS calculator. Their results showed that age (IRR = 1.01, 95% CI: 1.01–1.01), ASA PS classification (IRR = 1.22, 95% CI: 1.20–1.24), and operative duration (IRR = 1.18, 95% CI: 1.18–1.18) were significant factors associated with prolonged LOS. Furthermore, postoperative complications such as deep incisional surgical site infection (IRR = 3.58, 95% CI: 3.39–3.78), superficial surgical site infection (IRR = 3.48, 95% CI: 3.30–3.66), septic shock (IRR = 2.17, 95% CI: 2.06–2.29), pneumonia (IRR = 2.01, 95% CI: 1.95–2.07), pulmonary embolism (IRR = 1.76, 95% CI: 1.65–1.80), and deep vein thrombosis (IRR = 1.75, 95% CI: 1.65–1.85) were also highly associated with significantly extended LOS. Additionally, that study developed a formula for calculating LOS using the beta coefficients of significant variables (8).

The findings of our study are largely consistent with those of Rhee et al. and Chona et al. collectively confirming that advanced age, longer operative duration, and higher ASA PS classification are core factors influencing LOS in orthopedic patients. This suggests these factors possess robust predictive value across different surgical types and populations. However, several differences exist in terms of study design, patient population, and statistical modeling. Regarding sample and design, our study specifically focused on patients undergoing orthopedic surgery with Class I incisions, complementing the populations studied by Rhee et al. (focused exclusively on joint arthroplasty) and Chona et al. (encompassing various orthopedic trauma procedures). By excluding the confounding effects of wound contamination, our study more precisely delineates the unique risk profile specific to clean surgical cases. In terms of effect estimation, our study not only corroborated these common factors but also innovatively identified the independent impact of preoperative electrolyte levels (such as potassium, magnesium, and calcium) on LOS, a finding not systematically reported in previous large-scale studies. Regarding model development, our study integrated results from both linear and logistic regression to construct a personalized nomogram prediction tool, which, compared to the calculator developed by Chona’s team, offers a more intuitive visualization to aid clinical decision-making.

Based on the findings of this study, we propose the following clinical recommendations for the perioperative management of patients undergoing orthopedic Class I incision surgery: First, patients of advanced age, with high ASA PS scores, high surgical grade, and prolonged operative time should be identified as key monitoring populations. For these patients, an individualized enhanced recovery pathway should be implemented, focusing on strengthening perioperative infection prevention and control, maintaining electrolyte balance, and implementing nutritional status monitoring and support. Second, perioperative care should include systematic monitoring and maintenance of electrolytes such as potassium, magnesium, and calcium at the upper limit of the normal range, preferably achieved through personalized nutritional support. Third, elevated preoperative serum sodium should be regarded as a risk warning sign, necessitating a comprehensive assessment of the patient’s volume status and comorbidities. Finally, antibiotic use should strictly adhere to guidelines, with microbial specimen collection and drug sensitivity testing performed prior to administration to achieve targeted anti-infective therapy. While unnecessary combination therapy should be avoided, reasonable combination regimens may be considered for patients with clearly identified high-risk factors. Through such a multidimensional and individualized management strategy, it is expected that postoperative recovery will be optimized and the length of hospital stay effectively controlled.

## 5 Limitations

Current research on the relationship between electrolytes and bone health primarily focuses on bone mineral density assessment and the pathogenesis of osteoporosis. However, there remains a paucity of systematic investigations into the interrelationships among bone mineral density, osteoporosis, fracture severity, and fracture healing rate. It is noteworthy that fracture severity and healing rate are critical clinical indicators influencing LOS. The absence of bone mineral density and osteoporosis-related data in this study may impose certain limitations on the comprehensiveness of the research findings. Furthermore, our study has limitations including the omission of certain variables previously established as important, such as blood transfusion and specific postoperative complications, which may contribute to the slightly lower explanatory power of our model compared to some previous reports. Future research should adopt multi-center prospective designs that integrate more comprehensive perioperative variables along with skeletal health indicators to further improve the model’s accuracy and generalizability.

## 6 Conclusion

The developed nomogram accurately predicts prolonged hospitalization risk in orthopedic patients with Class I incisions, integrating key determinants including age, surgical complexity, physiological status, and electrolyte levels. This tool demonstrates robust performance and offers tangible clinical utility for optimizing resource allocation and guiding personalized perioperative management.

## Data Availability

The original contributions presented in the study are included in the article/Supplementary material, further inquiries can be directed to the corresponding authors.
