# Supplementary material for: Factors influencing length of stay in orthopedic Class I incision surgery: development and validation of a nomogram using 31,248 patient records
Source: Front Med (Lausanne). 2026 Jan 12;12:1689556. doi: 10.3389/fmed.2025.1689556 (PMC12833462; doi:10.3389/fmed.2025.1689556)
Supplement: Supplementary file 1 [file Supplementary_file_1.docx]

**
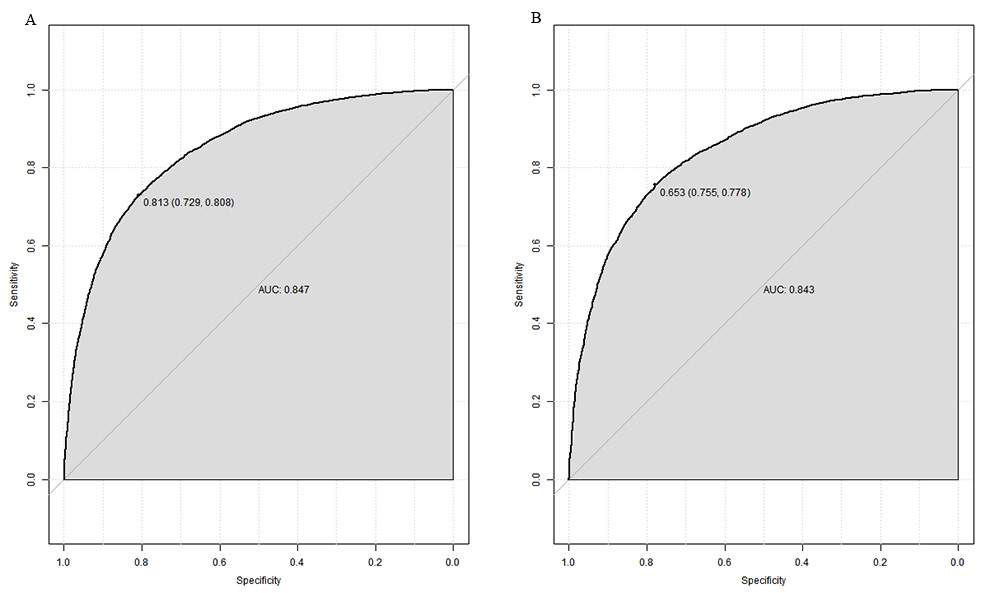
**

Figure 1 ROC curve of nomogram prediction model. (A) Training set. (B) Validation set. ROC, rreceiver operating characteristic; AUC, area under the ROC curve.

Training Set：AUC=0.847,95%CI：0.842-0.853

Validation Set：AUC=0.843,95%CI：0.835-0.851

**
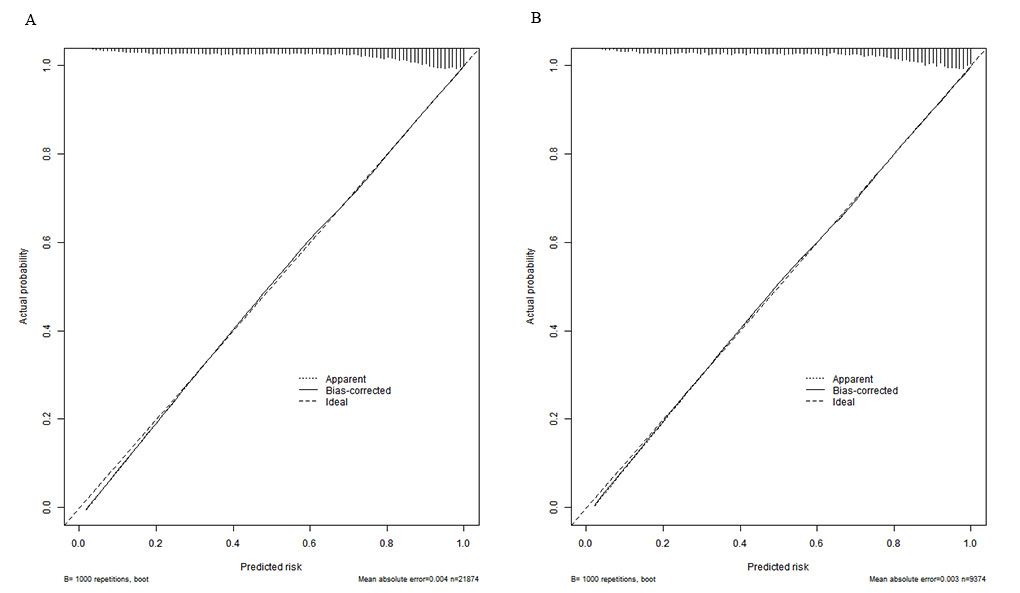
**

Figure 2 Bootstrap method validation nomogram curve. (A) Training set. (B) Validation set.

**
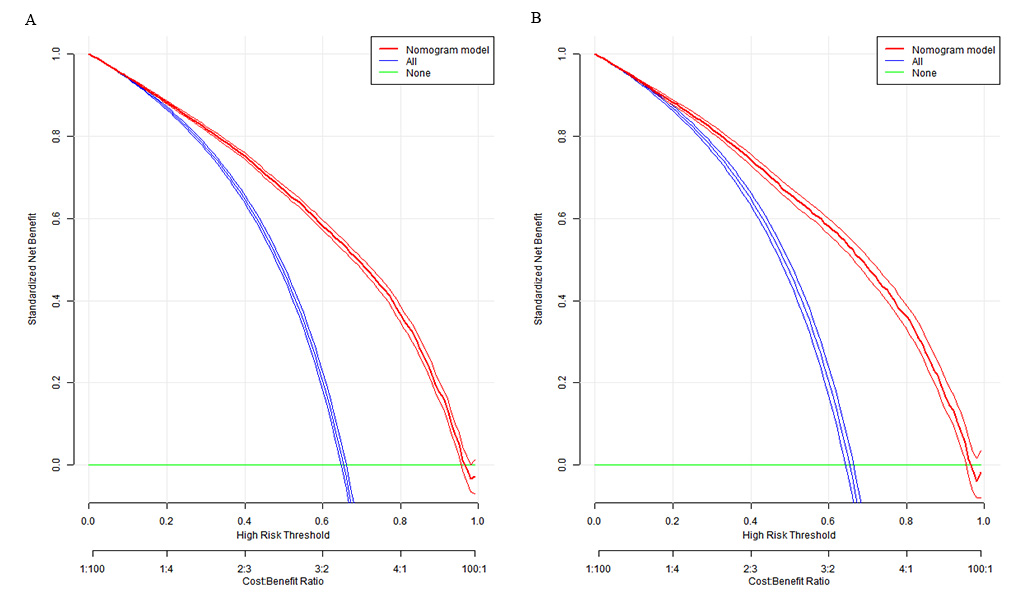
**

Figure 3 Clinical decision curve of nomogram model. (A) Training set. (B) Validation set.
