# Supplementary material for: Factors influencing length of stay in orthopedic Class I incision surgery: development and validation of a nomogram using 31,248 patient records
Source: Front Med (Lausanne). 2026 Jan 12;12:1689556. doi: 10.3389/fmed.2025.1689556 (PMC12833462; doi:10.3389/fmed.2025.1689556)
Supplement: Supplementary file 2 [file Table_1.docx]

Table 1 Statistical description of training set and validation set

| **Variables** | | **Mean ± SD / N (%)** | | ***t/χ^2^*** | ***P*** |
| --- | --- | --- | --- | --- | --- |
|  |  | **Training set**  **21874 (70.00)** | **Validation set**  **9374 (30.00)** |  |  |
| Gender | |  |  | 1.168 | 0.280 |
|  | Male | 9794（44.77） | 4135 (44.11) |  |  |
|  | Female | 12080（55.23） | 5239 (55.89) |  |  |
| Surgical grade | |  |  | 1.915 | 0.590 |
|  | 1 | 916（4.19） | 402（4.29） |  |  |
|  | 2  3333 | 9341（42.70） | 3927（41.89） |  |  |
|  | 3 | 6497（29.70） | 2836（30.25） |  |  |
|  | 4 | 5120（23.41） | 2209（23.57） |  |  |
| ASA PS | |  |  | 4.944 | 0.176 |
|  | 1 | 2489（11.38） | 1027（10.96） |  |  |
|  | 2 | 18022（82.39） | 7767（82.86） |  |  |
|  | 3 | 1285（5.87） | 559（5.96） |  |  |
|  | 4 | 78（0.36） | 21（0.22） |  |  |
| Antibiotic use | |  |  | 0.622 | 0.430 |
|  | No | 7772（35.53） | 3287（35.07） |  |  |
|  | Yes | 14102（64.47） | 6087（64.93） |  |  |
| Combination use of antibiotics | |  |  | 0.223 | 0.637 |
|  | No | 17294（79.06） | 7389（78.82） |  |  |
|  | Yes | 4580（20.94） | 1985（21.18） |  |  |
| Age | | 46.80 ± 19.38 | 47.18 ± 19.50 | 1.580 | 0.114 |
| Surgical duration (hour) | | 1.75 ± 1.23 | 1.75 ± 1.23 | 0.230 | 0.818 |
| K | | 4.05 ± 0.36 | 4.05 ± 0.36 | 0.271 | 0.786 |
| Na | | 141.27 ± 2.48 | 141.25 ± 2.89 | -0.518 | 0.604 |
| Mg | | 0.86 ± 0.09 | 0.85 ± 0.09 | -2.163 | 0.031 |
| P | | 1.11 ± 0.26 | 1.11 ± 0.26 | -0.769 | 0.442 |
| Ca | | 2.29 ± 0.13 | 2.29 ± 0.14 | -0.434 | 0.664 |

Note: SD: standard deviation; ASA PS: American Society of Anesthesiologists Physical Status Classification.
